# Donor Chimerism Study by Single Nucleotide Polymorphism using SYBR green based Real Time PCR

**DOI:** 10.12669/pjms.37.7.4203

**Published:** 2021

**Authors:** Ayesha Nayyar, Suhaib Ahmed

**Affiliations:** 1 Dr. Ayesha Nayyar, M.Phil. Department of Pathology, Islamic International Medical College, Riphah International University, Islamabad, Pakistan; 2 Prof. Dr. Suhaib Ahmed, FCPS, PhD. Department of Pathology, Islamic International Medical College, Riphah International University, Islamabad, Pakistan

**Keywords:** DC, SNP, STR, PCR, Informative allele, SYBR Green

## Abstract

**Objective:**

To optimize and evaluate a real time PCR of Single Nucleotide Polymorphism by SYBR Green method for detection of donor chimerism after haematopoietic stem cell transplantation.

**Methods:**

This descriptive study was conducted at Genetic Resource Centre (GRC) Lab Rawalpindi from Oct 2017 - Dec 2019. A total of twenty patients of post haematopoietic stem cell transplant with various haematological disorders were studied to see the status of donor chimerism by using SNP real time PCR using SYBR Green method and short tandem repeat PCR. These patients had undergone allogeneic HSCT from HLA-matched sibling donors at Pakistan Institute of Medical Science and Armed Forces Bone Marrow Transplant Centre.

**Results:**

Real time PCR using SYBR Green was able to detect significant amount of chimerism in all 20 patients having undergone HSCT. Regarding precision of the real time PCR assay the mean value of donor chimerism was 94.1% (SD 3.96) and by STR PCR it was 95.1% (SD 1.41). The assay was found to be sensitive with a detection limit of <1%.

**Conclusion:**

Our results demonstrate that SNP analysis by SYBR Green real time PCR may be used for the evaluation of chimerism status in patients having undergone HSCT with a sensitivity of <1%. Hence donor chimerism by this sensitive method can be used in monitoring of chimerism in post-transplant patients with various haematological disorders.

## INTRODUCTION

Haematopoietic stem cell transplantation (HSCT) has been extensively used for patients suffering from malignant hematological or nonmalignant hematological diseases,^1^ and considered as a treatment of choice for these patients over the past few decades^2^ The treatment failure in these patients may be due to relapse of the disease, graft rejection, and graft-versus-host disease (GVHD).^3^ Hence post-transplant monitoring in HSCT is used to predict relapse status, graft rejection and GVHD, in order to tailor the appropriate therapy.^4^

Inspection of chimerism status plays a pivotal role in monitoring of the patients with HSCT. The outcome of a stem cell transplant may be complete donor chimerism (100% donor cells) or mixed donor chimerism of varying proportion of donor and recipient cells.^5^ Early assessment of chimerism patterns may help to predict the recurrence or persistence of the disease as well as the bone marrow engraftment and rejection of the graft.^6^

There are various PCR based assays for the detection of chimerism status after HSCT. Single nucleotide polymorphisms (SNPs) are one of the common genetic variations^7^ and are considered as useful genetic markers for monitoring chimerism status after HSCT as these may be used to discriminate alleles between donor and recipient pairs.^8^ by detecting differences at single nucleotide bases and may be used as a molecular marker in PCR based assays.^9^

Currently the gold standard method for quantitative analysis of chimerism is STR-PCR on a genetic analyzer.^10^ The method, though sensitive, is costly. A cheaper alternative to the use of genetic analyzer is to do STR analysis on polyacrylamide gel electrophoresis (PAGE) and employing densitometry for quantitative assessment of the individual fractions. The STR-PCR using PAGE is less sensitive with high coefficient of variation.^11^ The problems which may be encountered are stutter peaks, which are artifacts due to polymerase slippages and these may interfere with the analysis of engraftment of the stem cell transplant.^5^ Moreover STR-PCR has more complex data analysis procedure that makes it more time consuming.^12^

According to Alizadeh et al real time PCR by hydrolysis probe method is the best method for assessing and evaluating the chimerism status.^2^ SNP analysis by real time PCR using the hydrolysis probes is expensive because each SNP marker requires a separate probe. The cost of SNP analysis can be reduced considerably if the use of hydrolysis probes is substituted by SYBR Green based detection of the amplified PCR product. There is no such study available locally which could help in formulating the role of real time PCR using SYBR Green dye for detection of donor chimerism in patients with various hematological disorders.

At present there are at least six centers in Pakistan that are doing HSCT for common haematological disorders like aplastic anaemia, thalassaemia and leukaemias. Most of these centers are using PAGE based manual methods for assessment of chimerism status. This research will provide a comprehensive methodology for sensitive and accurate assessment of donor chimerism status and will improve the standards of patient care after HSCT in Pakistan.

## METHODS

It was a descriptive study conducted at GRC Lab Rawalpindi. The study protocol was approved by institutional ethical review board, Faculty of Medical Sciences, RIU Islamabad-Pakistan (Ref. # Riphah/IIMC/IRC/20/135). The subjects of study were twenty post-transplant patients having undergone HSCT with various haematological disorders at PIMS and AFBMTC.

Approx 3-ml of venous blood was collected in EDTA. Genomic DNA was extracted from peripheral blood by Chelex™ method.^5^ The presence of informative SNP marker and STR locus by using eighteen biallelic SNPs and ten STR loci was searched in the peripheral blood samples of these patients before HSCT.

For PCR amplification of the STRs specific primers designed to flank repeated units of the different human gene regions were used as described by Ahmed.^5^ For SNP real time PCR a total of 18 human bi-allelic SNPs with high level of heterozygosity were selected as described by Alizadeh et al.^2^ Regarding donor chimerism of STR, each PCR was carried out in a 25 µl reaction mixture containing 10 pM of each primer, 0.5 units of Taq polymerase (Thermo Fisher, USA), 30 mM of each dNTP (Thermo Fisher, USA), 10 mM Tris HCl (pH 8.3), 50 mM KCl, 1.5 mM MgCl_2_ and 0.2µg of genomic DNA. Thermal cycling was done in Gene Amp 9700 (Applied Biosystems, USA) using a protocol of initial denaturation: five minutes at 95ºC with 30 Cycles each comprising Denaturation: 15 seconds at 95^º^C, Annealing: 60 seconds at 60ºC, Extension: 60 seconds at 72^º^C. The amplified products were loaded and analyzed on 6% polyacrylamide gel. The results of polyacrylamide gel by STR-PCR were photographed and were analyzed by the Thal-IT image analysis software (http://thal-it.com) in all twenty patients. The results of Donor Chimerism were expressed as percent of complete donor Chimerism

For the chimerism quantification by real time PCR of SYBR Green, two PCRs were run separately for the specific SNP marker and the reference gene (human GAPDH). The real time PCR amplification of each SNP and the GAPDH gene was done in a 20 µl reaction mixture (SolGent, Korea) containing PCR buffer, SYBR Green fluorescent dye, taq polymerase. The primer and the DNA concentration per reaction were the same as for the STR- PCR. The real time PCR amplification was done in a Rotor-Gene-Q machine (Qaigen, USA) using initial denaturation for 15 minutes at 95^º^C and 35 cycles comprising of denaturation 20 seconds at 95ºC, annealing/signal acquisition for 40 seconds at 60ºC and extension 60 seconds at 72ºC. At the end of the real time PCR the plot was examined for the cycle threshold (Ct) of each sample and the reference gene, GAPDH.

For calculation of donor Chimerism by real time PCR the Pfaffl method was used.^13^

ΔCt = Ct Donor Sample – Ct Recipient Post Transplant Sample

Ratio (R) = 2.0^ΔCt (SNP)^/ 2.0^ΔCt (GAPDH)^

The sensitivity of the SNP real time PCR assay was checked by running eight serial dilution of a single sample at GAPDH locus. For each dilution the respective Ct was recorded. The sensitivity and accuracy of quantitative SNP analysis by real time PCR was compared with the gel electrophoresis of STRs. To assess the effective performance of real-time PCR chimerism assay in post-transplant monitoring, a five repeat measures on the same sibling (donor/recipient) pair was also performed and assessed by the both methods.

## RESULTS

Results of donor chimerism by real time SNP PCR in the 20 sibling pairs were recorded in the form of PCR plots. The quantification of chimerism is summarized in Table-I. The level of chimerism ranged from 2.3% to 98.3%.

**Table I:** Comparison of the chimerism analysis by STR - PCR and real time SNP- PCR.

	*Chimerism Status*			

*Donor/Recipient Pairs*	*SNP - PCR*	*STR -PCR*	*Difference*
Pair 1	41.2	55.2	14.0
Pair 2	77.4	85.5	8.1
Pair 3	97.9	95.1	2.8
Pair 4	95.3	95.3	0
Pair 5	97.9	96.4	1.5
Pair 6	98.6	95.6	3.0
Pair 7	2.3	10.2	7.9
Pair 8	94.0	95.2	1.2
Pair 9	59.0	70.5	11.5
Pair 10	94.6	95.7	11.0
Pair 11	95.3	95.6	0.3
Pair 12	16.7	20.7	4.0
Pair 13	97.9	95.2	2.7
Pair 14	90.1	92.9	2.8
Pair 15	93.3	96.3	3.0
Pair 16	95.3	97.4	2.1
Pair 17	94.6	95.5	0.9
Pair 18	94.0	97.7	3.7
Pair 19	97.9	95.4	2.5
Pair 20	4.6	15.6	11.0

Results of donor chimerism by STR PCR in the 20 sibling pairs were read from the silver-stained polyacrylamide gels and their densitometry. The overall results of the 20 sibling pairs are summarized in Table-I. The level of chimerism ranged from 10.2% to 97.7%.

**Fig.1:**
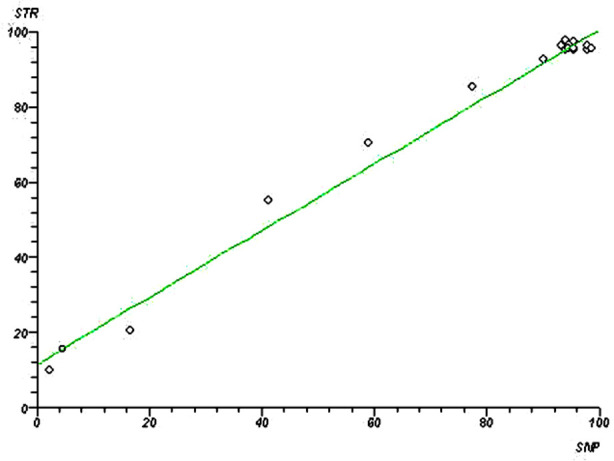
Simple linear regression analysis of the chimerism results between STR-PCR and real time SNP PCR.

The results of donor chimerism by real time SNP PCR and STR PCR in the 20 patients were compared (Table-I). The mean percent value obtained by real time SNP-PCR was 4.7% (95% CI 2.7-6.9%) less than that obtained by STR-PCR (*p* < 0.001). There was a good linear correlation between the donor chimerism results measured by real time SNP-PCR and the STR-PCR. The scatter plot between the percentage of donor DNA measured by STR-PCR against the percentage of donor DNA measured by the real time SNP-PCR showed an overall correlation coefficient (r) of 0.993173 and (r² = 0.986392) (Fig.1).

**Fig.2:**
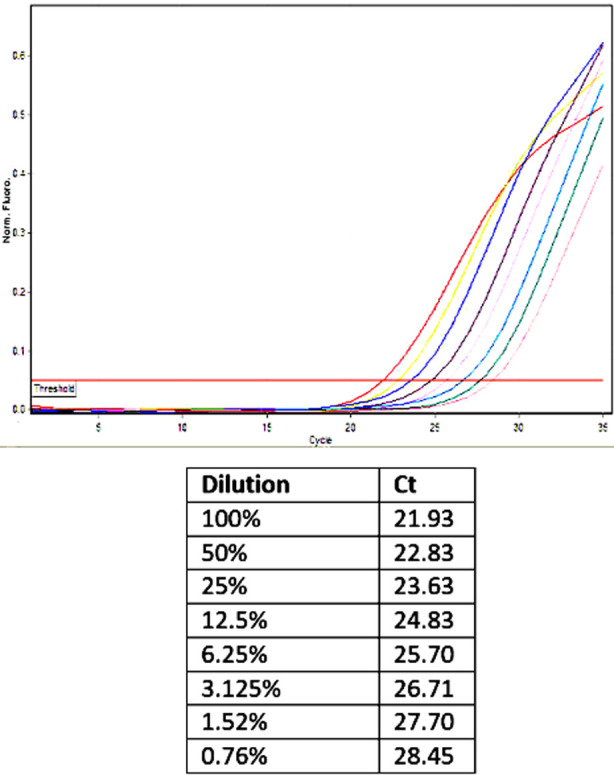
Real Time PCR plot of eight serial dilutions amplified at the GAPDH locus. The amplified product is clearly detectable at the final dilution of 0.76% (<1%)

A comparison of five repeat measures on the same sibling pair for the chimerism results between real time SNP PCR and STR PCR showed that the mean value of donor chimerism by real time SNP-PCR was 94.1% (SD 3.96) and by STR-PCR it was 95.1% (SD 1.41). Regarding sensitivity of the SNP-PCR assay, the results of the real time PCR plot and the Ct values are shown in Fig.2. The amplified product was clearly detectable at the final dilution of 0.8% (<1%).

## DISCUSSION

Chimerism analysis is considered an essential method in monitoring the post HSCT outcome in patients with different hematological disorders.^14^ Various assays have been developed to monitor the donor chimerism after HSCT. These methods include: Fluorescent in situ hybridization^15^, Real time PCR by Indel polymorphism^11^, real time PCR by STR^16^ or VNTRs^17^, and real time PCR by SNP analysis using hydrolysis or hybridization probes.^2^

All these methods are relatively laborious, need technical expertise and several days are generally required to perform these assays. Moreover, many PCR based techniques require the post PCR analysis which increase the turnaround time such as agarose/acrylamide gel electrophoresis, restriction enzyme digestion, or automated sequence analysis.^18^

A group of researchers have analyzed the chimerism status by using SNPs as a genetic marker for the assessment of chimerism pattern.^2^ Greinkie et al. concluded that SNPs can be used as a useful genetic marker in monitoring the chimerism status.^3^ Koldehoff et al. proved that real time PCR using SNPs plays an important role in determining the chimerism after HSCT.^19^

Our data have also suggested that SNP real-time PCR by using SYBR Green has a higher sensitivity than STR-PCR. A serial dilution of DNA amplified at GAPDH locus was tested to determine the sensitivity of the assay which was observed at 0.8%, making it more reproducible technique for detecting low amount of donor chimerism.

Weidermann et al.^20^ proposed that, real time PCR has greater sensitivity and linearity over STR-PCR. This higher sensitivity of SNP-PCR may be responsible for earlier detection of mixed chimerism in patients who may undergo relapse at later stages after HSCT. Few researchers have concluded that the real-time PCR having a sensitivity of up to 1x10^4^ is much more sensitive than the other methods for chimerism analysis e.g., STR-PCR and VNTR-PCR, which have a sensitivity of up to 1x10^3^ & 1x10^2^.^21^

Regarding donor chimerism, the results obtained for quantification with STR-PCR assay were quite similar with those achieved by real time SNP- PCR using SYBR Green. The precision of SNP real time PCR and STR-PCR was done with repeated measures on the same DNA samples and the results were quantified. The percentage chimerism for SNP real time PCR was 94.9±3.96 and for STR-PCR it was found to be 95.1±1.41. This precision appears to be quiet significant for a practical use in clinical situations in patients who may undergo HSCT. Due to high discrimination ability, high accuracy and precision of real time SNP-PCR and STR-PCR, both of these methods remain probably crucial for chimerism analysis after HSCT.

Ling et al.^12^ conducted a study on 57 paediatric patients for analysis of chimerism status by using SNP real time PCR and STR-PCR and concluded that any of these methods can be used for the assessment of chimerism analysis. Koldehoff et al.^19^ studied 135 patients of hematopoietic stem cell transplantation by real time PCR of SNPs and STR-PCR and found out that SNP-PCR has much higher sensitivity as compared to the later one and should be used in routine practice for assessing the donor chimerism.

A group of researchers in their study have stated that SYBR green by SNP-PCR could be used as an alternative to hydrolysis probes or hybridization probes technology in real- time PCR for the detection of genetic variation.^21^

Few researchers stated that SYBR Green, a DNA-intercalating agent, can be used in real-time PCR methods as a fluorescent label.^22^ Almaieda et al.^23^ conducted a study in 88 donor/recipient pairs, by real time PCR using SYBR green of eight SNPs and found that these may be used further in detecting donor chimerism. A group of researchers concluded that the quantitative real time PCR using twelve SNPs by SYBR Green was able to differentiate between recipient and donor genetic patterns in all of the 18 samples studied and hence capable of quantifying the chimerism in all these samples.^24^

Donor chimerism detection by STR-PCR and SNP-PCR based assay is quite feasible in our set up in terms of materials availability, technical expertise. The test is fairly simple and can be done at any laboratory equipped to do PCR. The cost of DNA extraction and real time PCR by SYBR Green method costs Rs/400 per reaction that is much cheaper than the real time PCR by hydrolysis probe method as each probe costs Rs.32,000. Comparing the, rapidity and practicability of the two methods of chimerism analysis, STR-PCR is more time consuming than the real time SNR-PCR because of the additional time for gel electrophoresis of the amplified PCR products in STR-PCR. Moreover, real time PCR provides the opportunity of giving the final results within two hours making it more practicable for use in clinical practice.

### Limitation of the study

It includes small sample size. However, the findings provide a basis for carrying further studies.

## CONCLUSION

This study has introduced a cost-effective real time SNP-PCR based method of assessing donor chimerism status in patients undergoing HSCT in Pakistan. The method is simple, quick, sensitive, and accurate. The combination of SNPs and SYBR Green enables simple and accurate analysis of donor chimerism without using expensive fluorescent primers. The procedure requires a general real time PCR apparatus, SYBR Green dye and PCR mixture in a single PCR tube. It would be a convenient tool for the clinical researchers to analyze donor chimerism through this simple and cost effective method.

### Authors’ Contribution:

**AN:** Literature search, study design and concept, data collection, data analysis, data interpretation, discussion, drafting. **SA:** Study design and concept, data analysis, data interpretation, Critical Review, Final approval.
